# Construction and Comprehensive Analyses of a METTL5-Associated Prognostic Signature With Immune Implication in Lung Adenocarcinomas

**DOI:** 10.3389/fgene.2020.617174

**Published:** 2021-02-19

**Authors:** Sijin Sun, Kailun Fei, Guochao Zhang, Juhong Wang, Yannan Yang, Wei Guo, Zhenlin Yang, Jie Wang, Qi Xue, Yibo Gao, Jie He

**Affiliations:** ^1^Department of Thoracic Surgery, National Cancer Center/National Clinical Research Center for Cancer/Cancer Hospital, Chinese Academy of Medical Sciences and Peking Union Medical College, Beijing, China; ^2^State Key Laboratory of Molecular Oncology, Department of Medical Oncology, National Cancer Center/National Clinical Research Center for Cancer/Cancer Hospital, Chinese Academy of Medical Sciences and Peking Union Medical College, Beijing, China; ^3^State Key Laboratory of Molecular Oncology, National Cancer Center/National Clinical Research Center for Cancer/Cancer Hospital, Chinese Academy of Medical Sciences and Peking Union Medical College, Beijing, China

**Keywords:** rRNA methylation, prognosis, *METTL5*, machine learning, lung adenocarcinoma

## Abstract

For lung adenocarcinoma (LUAD), patients of different stages have strong heterogeneity, and their overall prognosis varies greatly. Thus, exploration of novel biomarkers to better clarify the characteristics of LUAD is urgent. Multi-omics information of LUAD patients were collected form TCGA. Three independent LUAD cohorts were obtained from gene expression omnibus (GEO). A multi-omics correlation analysis of *METTL5* was performed in TCGA dataset. To build a *METTL5*-associated prognostic score (MAPS). Spathial and random forest methods were first applied for feature selection. Then, LASSO was implemented to develop the model in TCGA cohort. The prognostic value of MAPS was validated in three independent GEO datasets. Finally, functional annotation was conducted using gene set enrichment analysis (GSEA) and the abundances of infiltrated immune cells were estimated by ImmuCellAI algorithm. A total of 901 LUAD patients were included. The expression of *METTL5* in LUAD was significantly higher than that in normal lung tissue. And high expression of *METTL5* indicated poor prognosis in all different stages (*P* < 0.001, HR = 1.81). Five genes (*RAC1, C11of24, METTL5, RCCD1*, and *SLC7A5*) were used to construct MAPS and MAPS was significantly correlated with poor prognosis (*P* < 0.001, HR = 2.15). Furthermore, multivariate Cox regression analysis suggested MAPS as an independent prognostic factor. Functional enrichment revealed significant association between MAPS and several immune components and pathways. This study provides insights into the potential significance of *METTL5* in LUAD and MAPS can serve as a promising biomarker for LUAD.

## Introduction

As the world’s highest incidence and highest mortality, lung cancer causes more than 700,000 deaths each year (Bray et al., 2018). More than 40% of patients are lung adenocarcinoma (LUAD) (Lortet-Tieulent et al., 2014). Especially for the Asian population, the proportion of adenocarcinoma is further increased among non-smokers (Nakamura and Saji, 2014). Tumor prognosis of different stages is different for patients with LUAD, no matter which stage of the tumor, there is a greater heterogeneity in response to treatment and prognosis (Chen et al., 2014). Therefore, identification of patients with favorable response and relatively favorable prognosis is of great help in guiding treatment. At the same time, for advanced LUAD, although there are several measures including chemotherapy, targeted therapy and rapid development of immunotherapy in recent years, the 5-year survival rate of advanced LUAD is still less than 25% (Gandhi et al., 2018; Gettinger et al., 2018; Soria et al., 2018). Finding effective prognostic factors or therapeutic targets are urgent for patients with LUAD.

With the development of gene sequencing technology including next-generation sequencing, an increasingly deeper understanding in tumorigenesis and tumor development has been achieved. In addition to DNA mutations, gene expression levels or corresponding regulatory mechanisms will also impact the treatment and prognosis of tumors (Tano and Akimitsu, 2012; Dai et al., 2018). The modification of RNA by N6-methyladenosine (m6A) is an important mechanism regulating RNA function, especially mRNA function.

In previous studies, several m6A modification genes have been reported to involve in tumorigenesis and prognosis, which included *METTL3, METTL4* that promote m6A methylation (Lin S. et al., 2016; Chen et al., 2020), the gene that mediates demethylation of FTO (Li et al., 2017), and *YTHDF* family of genes that mediate m6A recognition and affect mRNA expression levels. However, the abovementioned regulatory mechanisms are mainly based on mRNA. It is not fully understood the modification function of another large class of RNA, ribosomal RNA. In an in-house screening for biomarkers, *METTL5* was shown to have great prognostic potential and was rarely reported before. Previous study suggested *METTL5* was involved in the regulation of methylation of 18srRNA (Liu et al., 2020). However, the biological function and prognostic implication of *METTL5* in tumors including LUAD remain unclear.

To our knowledge, this is the first study that comprehensively explored *METTL5* in LUAD. In the present study, we downloaded and re-analyzed a total of 901 samples from The Cancer Genome Atlas cohort (TCGA-LUAD) and gene expression omnibus (GEO) database. Multi-omics profiles and survival analysis of METTL5 were performed. In order to enhance the predictive efficacy, a *METTL5*-centered prognostic signature was constructed in TCGA-LUAD by implementing several machine learning algorithms. The signature was further validated and evaluated in three independent cohorts. Furthermore, functional annotation of the signature was conducted and immune implication of the signature was explored by estimating abundances of immune cells between different subgroups.

## Materials and Methods

### Data Acquisition and Preprocessing

The multi-omics LUAD dataset including mRNA expression profile, DNA methylation, gene mutation and copy number variation (CNV) data was retrieved from the Cancer Genome Atlas (TCGA) Data Portal (^1^ May, 2020). For TCGA-LUAD cohort, duplicated samples were first removed and only samples with transcriptome data and complete survival information were included for model training. For validation cohorts, three microarray datasets were obtained from GEO. GISTIC2 method (Mermel et al., 2011) and beta value were applied to quantify CNV and methylation status, respectively. And transcripts per million (TPM) was calculated from Fragments Per Kilobase Million (FPKM) for RNA-Seq data. The Human Protein Atlas^2^ was employed to display the distribution and intensity of the *METTL5* protein in LUAD and normal lung tissue.

### Construction of a *METTL5*-Associated Prognostic Signature in TCGA-LUAD

To assess the prognostic significance of METTL5, Cox regression and Kaplan–Meier survival analysis were conducted in TCGA-LUAD cohort and in subgroups stratified by pathologic stage. The optimal threshold for grouping was determined by the maximally selected rank statistics from the R package *maxstat* (Hothorn and Hothorn, 2007). To develop an effective predictive signature that was closed related to *METTL5*, a novel evolutionary analysis implemented in the R package *Spathial* was first applied to find genes associated with different expression level of *METTL5* (Gardini et al., 2020). Spathial employed Principal Path algorithm to find the most important genes involved in a certain process. Samples were stratified into two groups according to the upper and lower quantiles of METTL5 expression. The starting and ending points of the path were set as centroids of the two groups. During the path analysis, the number of waypoints was set to 50. Gene significances were ranked by adjusted *P* value and the top 100 genes were selected. Next, the random survival forest was employed to further reduce the number of features by R package *randomForestSRC* (Ishwaran et al., 2008). The training was running with “importance = TRUE, block size = 1” and set with all other parameters set to default. The top 15 genes ranked by variable importance (VIMP) were included for penalized regression analysis. Ultimately, the *METTL5*-associated prognostic signature (MAPS) was constructed through least absolute shrinkage and selection operator (LASSO) regression and the optimal parameter was determined through 10-fold cross validation (Tibshirani, 1997) with “family = cox, first element of penalty factor = 0” and with all other parameters set to default. The MAPS was calculated with the following formula: $\int_{i\,1}^{n}\beta_{i}\,G_{i}$, where β_*i*_ represents the coefficient of gene *i*, *G_i* is the normalized expression value of gene *i.*

### Validation of MAPS in Independent Datasets

To validate the predictive value of MAPS, Cox regression and Kaplan–Meier survival analysis were performed in three independent cohorts (GSE3141, GSE13213, and GSE31210) (Bild et al., 2006; Tomida et al., 2009; Okayama et al., 2012). MAPS was calculated for each sample by the formula developed in TCGA-LUAD. The median of MAPS served as the cutoff value for stratification. Further, since three datasets contained more complete clinical information, multivariate Cox regression model was implemented to control confounders.

### Functional Enrichment Analyses of *METTL5* and MAPS

To explore the potential biological role of *METTL5*, the STRING web server (version 11.0) was first used to construct a *METTL5*-centered protein-protein interaction network (Szklarczyk et al., 2019). The minimum interaction score was set to 0.4. Gene Ontology (GO) and Kyoto Encyclopedia of Genes and Genomes (KEGG) pathways were employed for functional annotation of the network. Next, in order to understand the potential biological relevance of MAPS, gene set enrichment analysis (GESA) was conducted based on the R package *clusterProfiler* between different risk groups (Yu et al., 2012). The BIOCARTA subset of canonical pathways was used and obtained from the Molecular Signatures Database (version 7.2) (Liberzon et al., 2011).

### Assessment of Tumor Microenvironment and Infiltrated Immune Cells

The tumor microenvironment was calculated using ESTIMATE algorithm and two scores including stromal score and immune score were retrieved for each TCGA-LUAD samples (Yoshihara et al., 2013). In addition, Immune Cell Abundance Identifer (ImmuCellAI) was applied to estimate the abundance of 24 immune cell types including 18 T-cell subclasses (Miao et al., 2020). Abundances of each cell type were compared between different risk groups.

### Calculation of Tumor Mutation Burden in TCGA-LUAD Cohort

Tumor mutational burden (TMB) is a measurement of the number of gene mutations carried by cancer cells. In the present study, TMB was defined as the total number of non-synonymous mutations in whole-exome genomic region. After calculating TMB for each sample, the comparison between low-risk and high-risk groups was performed.

### Comparisons of MAPS and an m6A-Prognostic Signature in Multiple Cohorts

To provide further evidence for the clinical value of the constructed gene model, MAPS was then compared to a published model with similar biological function. A m6A-prognostic signature reported by Li et al. were extracted and risk score was calculated for each sample according to the provided formula in all included cohorts except GSE13213 due to lack of gene probes. The prognostic value of these two models was quantified and compared according to the concordance index (C-index). The restricted mean survival (RMS) curve was used to present the life expectancy of different risk groups at 120 months. A larger slope of the RMS curves indicated a superior predictive performance.

### Statistical Analysis

All statistical analyses were conducted using R version 3.6.2. For continuous variables, Wilcoxon test and Kruskal–Wallis test were applied to compare two groups and multiple groups, respectively. Pearson’s correlations were employed to assess associations between two variables. Both Cox regression and Log-rank test were selected for survival analysis. Receiver operating characteristic (ROC) curve was used to evaluate predictive efficacy by *plotROC* package (Sachs, 2017). C-index was calculated and compared using *survcomp* and *compareC* packages, respectively (Kang et al., 2015). A *P* value less than 0.05 was considered statistically significant.

## Results

### Multi-Omics Profiles of *METTL5* in TCGA-LUAD Cohort

**FIGURE 1:**
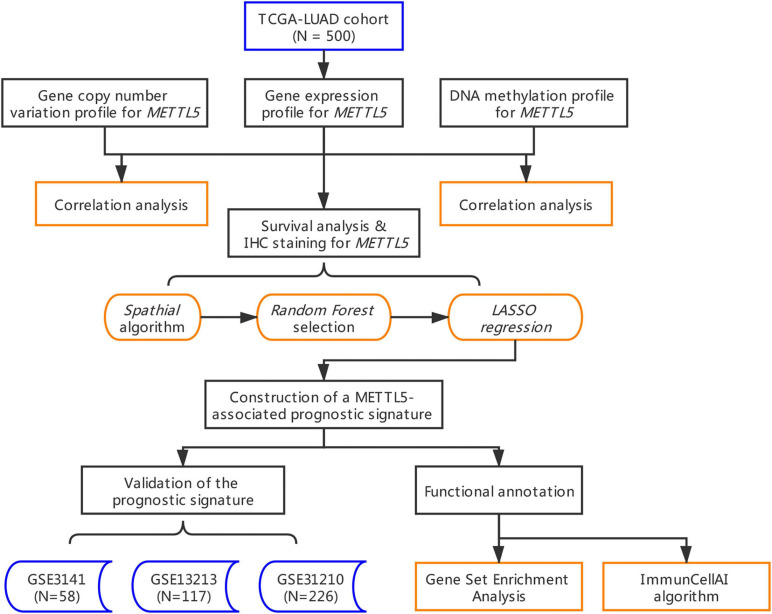
Flowchart of the whole study.

**FIGURE 2:**
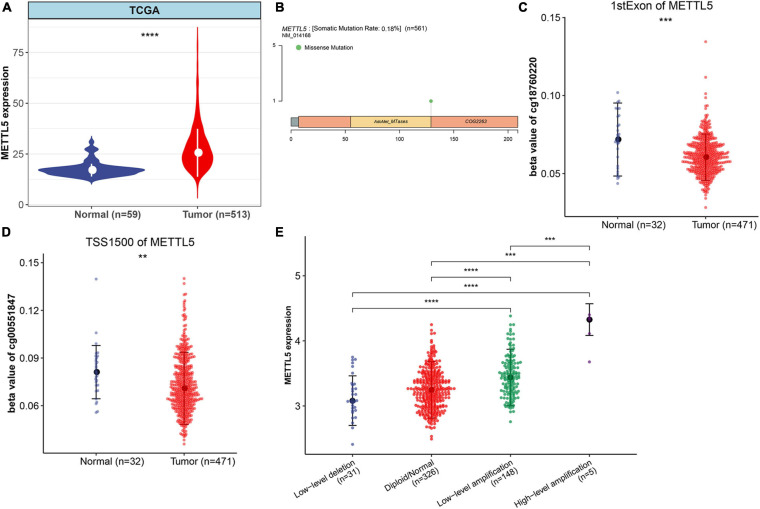
Multi-omics profiles and association analysis of *METTL5* in TCGA-LUAD cohort. **(A)** Differential expression of *METTL5* between LUAD and normal adjacent lung tissue. **(B)** Mutation location and frequency of *METTL5*. **(C)** Methylation level of *METTL5* within the first exon between LUAD and normal adjacent lung tissue. **(D)** Methylation level of *METTL5* within TSS1500 between LUAD and normal adjacent lung tissue. **(E)** Association analysis between *METTL5* expression and CNV. The expression level and CNV were quantified using log (TPM+1) and GISTIC2 score, respectively. *p < 0.05; **p = 0.01; ***p = 0.001; ****p < 0.0001.

The whole workflow and key steps were depicted in Figure 1. Compared to the adjacent normal lung tissue, *METTL5* expression was significantly (*P* < 0.001) upregulated in LUAD (Figure 2A). To further depict the possible mechanisms associated with *METTL5* overexpression, mutational profile of *METTL5* was first display and only one missense mutation was found (Figure 2B). Further, the methylation levels of *METTL5* at 1st Exon (*P* < 0.001) and TSS1500 (200–1,500 nt upstream of transcription start sites) (*P* = 0.004) was significantly lower in LUAD (*n* = 370) than that in normal lung tissues (Figures 2C,D). In addition, significant association of between *METTL5* expression and CNV was found in LUAD (Figure 2E). This together suggested that *METTL5* expression was partially influenced by DNA methylation and CNV.

### Prognostic Value and Functional Annotation of *METTL5*

**FIGURE 3:**
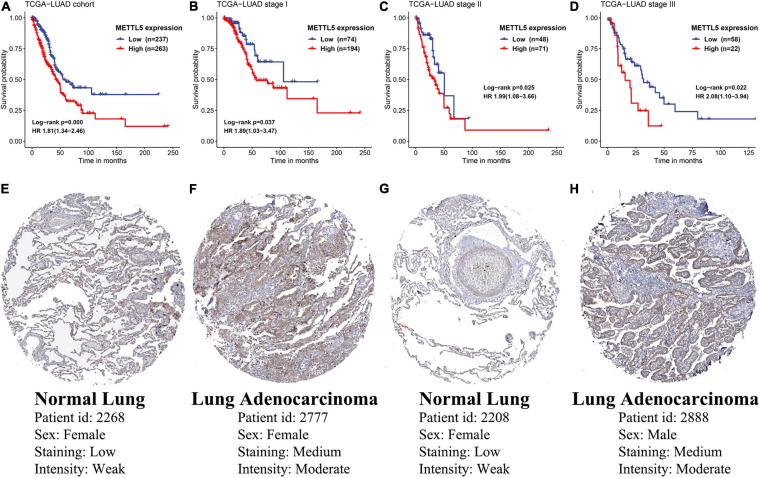
Prognostic significance and protein-level expression of *METTL5*. Kaplan–Meier survival curves and univariate Cox regression of OS in **(A)** the whole TCGA-LUAD cohort, **(B)** stage I subgroup, **(C)** stage II subgroup, and **(D)** stage III subgroup. Representative immunohistochemical staining of *METTL5* in normal lung tissue **(E,G)** and LUAD **(F,H)**.

**FIGURE 4:**
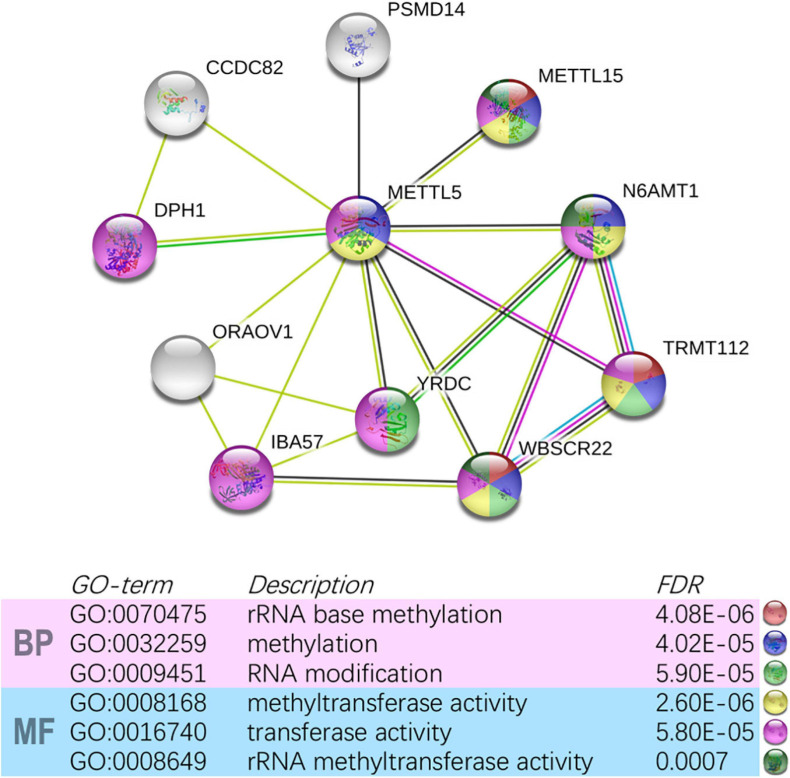
Construction of *METTL5*-centered protein-protein interaction network and functional annotation using STRING.

In TCGA-LUAD cohort, a total of 500 samples with complete transcriptome data and survival information were included. Both Kaplan–Meier survival curve and Cox regression revealed that patients with high expression of *METTL5* had a significantly (*P* < 0.001) worse prognosis than those with low expression of *METTL5* (Figure 3A). In addition, the overall survival (OS) time was significantly shorter in patients with high *METTL5* expression than those with low *METTL5* expression in stage I, stage II and stage III subgroups (Figures 3B–D). To further illustrate the clinical utility of *METTL5*, typical immunohistochemical staining images of *METTL5* in both LUAD and adjacent normal lung tissue were shown in Figures 3E–H. Based on the results from STRING database, *METTL5* was closely related to ten genes and functional enrichment analysis suggested that they were significantly enriched in rRNA base methylation, methylation, RNA modification, methyltransferase activity, transferase activity, and rRNA methyltransferase activity (Figure 4).

### Construction of a *METTL5*-Associated Prognostic Model in TCGA-LUAD Cohort

**FIGURE 5:**
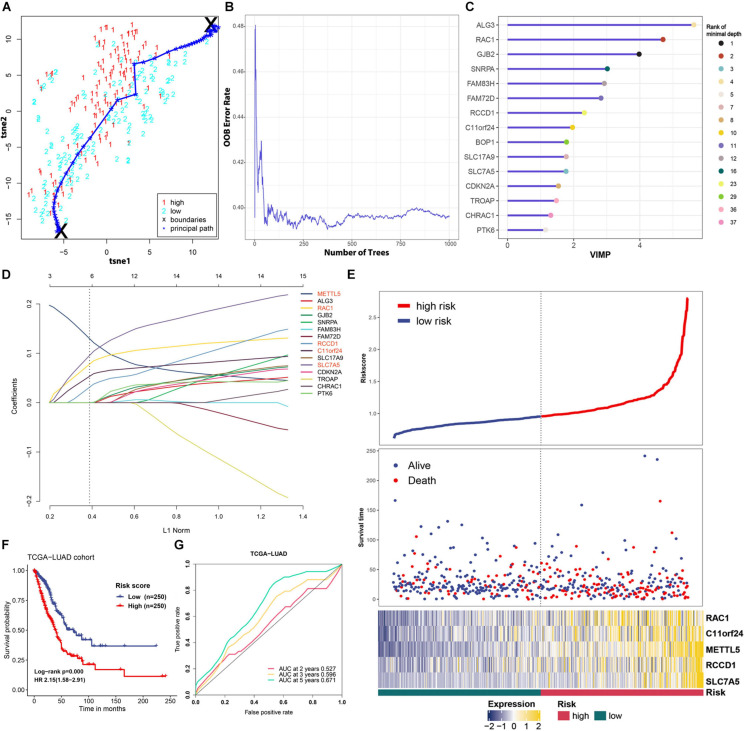
Feature selection and prognostic model construction process. **(A)** 2D visualization of the principal path and data points in TCGA-LUAD cohort. Red and blue points represent high-level expression and low-level expression of *METTL5*, respectively. **(B)** Error plot of the random survival forest model quantified by OOB prediction error. **(C)** The top 15 genes ranked by VIMP in the random survival forest. The ranking according to minimal depth was also presented using different color points. **(D)** Construction of the final prognostic model using LASSO Cox regression. Names of included genes were labeled in red. **(E)** The distribution of survival status and expression profile of selected genes between different risk groups. **(F)** Survival analysis of MAPS using Kaplan–Meier survival curves and univariate Cox regression in TCGA-LUAD cohort. **(G)** Prognostic value of MAPS evaluated by ROC curves in TCGA-LUAD cohort.

To improve the prognostic ability of *METTL5* further, a signature named MAPS was constructed in TCGA-LUAD cohort. The Principal Path algorithm was first applied to identify *METTL5* associated genes and the selected path together with data points was visualized using a dimensionality reduction manner (Figure 5A). The top 100 genes were selected as input to random survival forests and a full list of these genes were append to Supplementary Table 1. The error plot quantified by the Out-of-Bag (OOB) error rate demonstrated that the prediction accuracy was getting stable when the number of trees reached around 375 (Figure 5B). To reduce the number of useless features, the top 15 genes were selected according to their VIMP and the corresponding rank with minimal depth was illustrated in Figure 5C. Next, LASSO regression was performed using 10-fold cross validation on these selected genes and the optimal λ was set to 0.038. Ultimately, five candidate genes, including *METTL5*, RAC1, RCCD1, C11orf24, and SLC7A5, were included in the final signature. The MACS was calculated as followed: (0.130 × EXP*METTL5*) + (0.078 × EXPRAC1) + (0.031 × EXPRCCD1) + (0.053 × EXPC11orf24) + (0.096 × EXPSLC7A5).

MACS of each cases in TCGA-LUAD cohort was calculated and patients were stratified into low-risk and high-risk groups according to the median. The distribution of survival status and expression profile of selected genes between subgroups was shown in Figure 5E. Kaplan–Meier survival analysis revealed that the OS time in high-risk group was significantly (*P* < 0.001) shorter than the low-risk group (Figure 5F). In addition, univariate Cox regression suggested that MAPS was a significant risk factor (HR = 2.15, *P* < 0.001) for survival (Figure 5F). ROC curves were employed to evaluate the predictive power and area under the curves (AUCs) for 2-, 3-, and 5-year OS were 0.527, 0.596 and 0.671, respectively (Figure 5G).

### Validation of MAPS in Three Independent Cohorts

**FIGURE 6:**
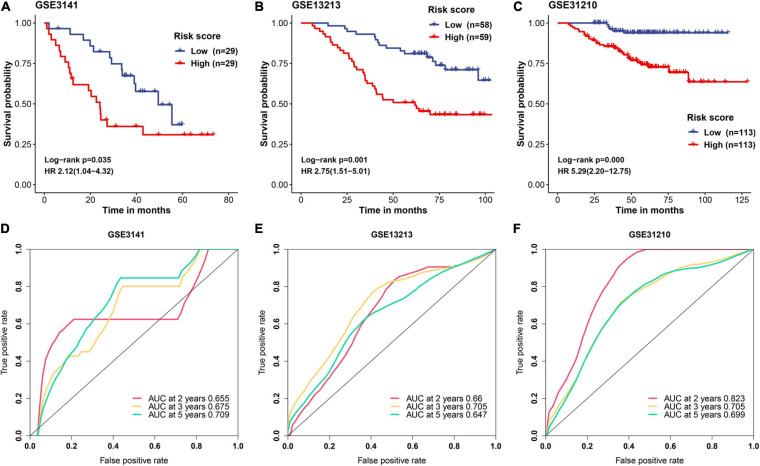
Validation of MAPS in three independent cohorts. Kaplan–Meier survival curves and univariate Cox regression of MAPS in **(A)** GSE3141, **(B)** GSE13213, and **(C)** GSE31210. Prognostic value evaluation by ROC curves in **(D)** GSE3141, **(E)** GSE13213, and **(F)** GSE31210.

A detailed description of three GEO cohorts was listed in Table 1. Within each cohort, MAPS was first calculated and samples were divided into low-risk and high-risk groups based on the median value of risk scores. The Kaplan–Meier survival curve suggested that the OS was significantly (*P* < 0.05) worse in the high-risk group than that in the low-risk group in all three cohorts (Figures 6A–C). Furthermore, univariate Cox regression revealed that MAPS was a significant risk factor (*P* < 0.05) for OS in all three cohorts (Figures 6A–C). ROC curves were used to assess the predictive utility and AUCs were ranging from 0.647 to 0.823 (Figures 6D–F). It was noteworthy to mention that MAPS present a promising prediction capability in a early-stage LUAD cohort (Figure 6F), which demonstrated its potential to further stratify LUAD in an early stage.

**TABLE 1:** Description of validation cohorts used in this article.

Author	Published year	Accession	Platform	Number of cases
Bild et al.	2006	GSE3141	GPL570	58
Tomida et al.	2009	GSE13213	GPL6480	117
Okayama et al.	2011	GSE31210	GPL570	226

### Identification of MAPS as an Independent Prognostic Factor

**FIGURE 7:**
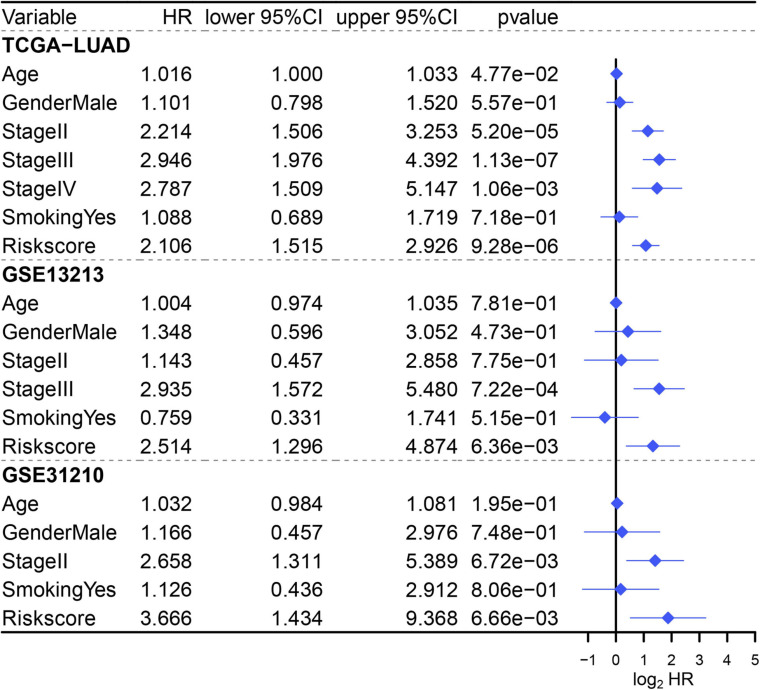
Multivariate Cox regression analysis of MAPS in three cohorts. Factors include age, gender, smoking history, stage, and MAPS.

Since three cohorts (TCGA-LUAD, GSE13213, and GSE31210) had complete clinical information, multivariate Cox regression analysis was conducted in these datasets to further illustrate the prognostic significance of MAPS. The results demonstrated that MAPS was an independent prognostic factor (*P* < 0.05) in all three datasets (Figure 7). The hazard ratios for OS were 2.106, 2.514, and 3.666 in TCGA-LUAD, GSE13213, and GSE31210, respectively. Survival analysis between different LUAD subtypes was conducted and both log-rank text and Cox regression analysis suggested LUAD subtype was not a significant prognostic factor (Supplementary Figure 1).

### Functional Enrichment Analysis of MAPS and Its Immune Implication

**FIGURE 8:**
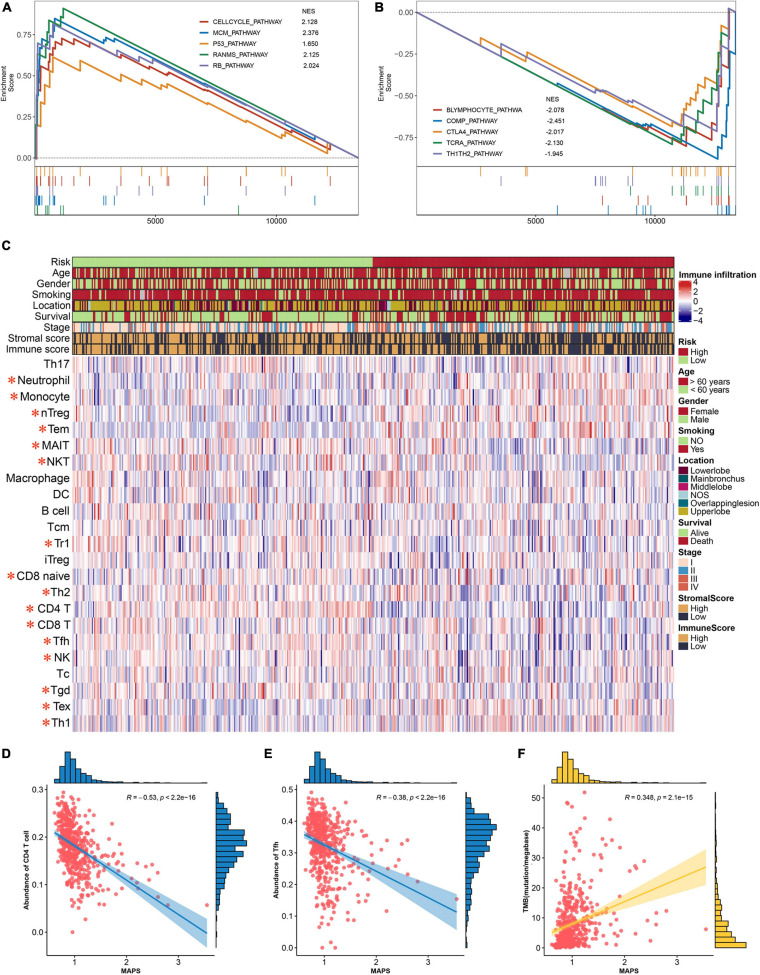
Functional annotation and immune implication of MAPS. **(A)** Significant enriched pathway in high-risk subgroup identified by GSEA. **(B)** Significant enriched pathway in low-risk subgroup identified by GSEA. **(C)** Profile of 24 infiltrated immune cells in different risk groups. The risk group, age, gender, smoking history, tumor location, survival status, tumor stage, stromal score, and immune score were used as annotation. Immune cells labeled in red are significantly differentially expressed. Correlation analysis between MAPS and **(D)** CD4 T cell, **(E)** Tfh, and **(F)** TMB. Data distribution of x and y axes were shown on the top and right side of each image.

To gain insights into the biological function of the MAPS, GSEA was performed using BIOCARTA gene set between low-risk and high-risk groups stratified by the risk score. A full list of significant enriched pathway was appended to Supplementary Table 2. The results revealed that CELLCYCLE pathway, MCM pathway, P53 pathway, RANMS pathway and RB pathway was significantly (*P* < 0.05) enriched in the high-risk group (Figure 8A). Surprisingly, several immune-related pathways were significantly (*P* < 0.05) correlated with low-risk group including B lymphocyte pathway, COMP pathway, CTLA4 pathway, TCRA pathway and TH1TH2 pathway (Figure 8B). Thus, abundances of infiltrated immune cells and microenvironment were next evaluated using ImmunCellAI and ESTIMATE algorithm, respectively. The abundance profile of 24 immune cells was shown in Figure 8C and Two-thirds of immune cells (16/24) were significantly different between two groups. Two of the most significant cells, CD4 T cell and Tfh, were selected to perform correlation analysis with MAPS and the Pearson correlation coefficients were −0.53 and −0.38, respectively (Figures 8D,E). Furthermore, MAPS was also significantly (*P* < 0.001) associated with TMB, which was a validated indicator for immunotherapeutic response. This together suggested a widely immune implication between different MAPS groups.

### Comparisons of MAPS and a Published m6A Prognostic Signature for LUAD

**FIGURE 9:**
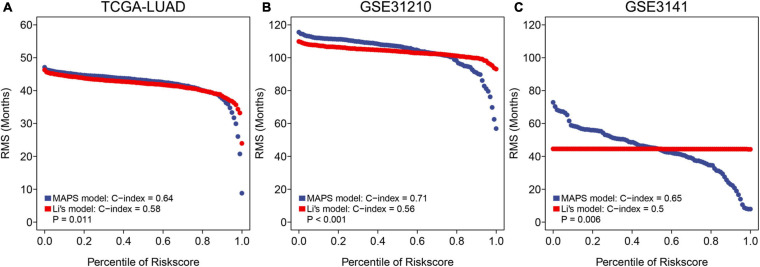
RMS curves and comparisons between MAPS and Li’s model in **(A)** TCGA-LUAD cohort, **(B)** GSE31210, and **(C)** GSE3141. The slope of MAPS curve was greater than Li’s curve in all three datasets, which indicated a superior predictive performance.

To further assess the predictive ability of MAPS, RMS curve and C-index were implemented to compare the performance of MAPS and a published m6A prognostic signature for LUAD. Significant improvement of the predictive efficacy was observed with MAPS relative to the m6A signature in TCGA-LUAD cohort (C-index: 0.64 vs. 0.58, *P* = 0.011, Figure 9A), GSE31210 (C-index: 0.71 vs. 0.56, *P* < 0.001, Figure 9B) and GSE3141(C-index: 0.65 vs. 0.50, *P* = 0.006, Figure 9C). The slope of MAPS curve was also greater than Li’s curve in all three datasets, which further proved the abovementioned conclusion.

## Discussion

In our research, the clinical and prognostic effects of *METTL5* was analyzed in LUAD based on the TCGA database, and further we developed a *METTL5*-based signature MAPS, which could effectively predict the prognosis of patients with LUAD. For the first time, multi-omics profile of *METTL5* was presented and the results suggested that the proportion of *METTL5* mutations was very low. DNA Methylation level and CNV amplification is closely related to *METTL5* expression. In addition, *METTL5* expression is a significant prognostic factor regardless of tumor stages. In order to achieve a better prediction accuracy, we constructed a molecular signature named MAPS including five genes based on *METTL5*. Furthermore, MAPS was significantly related to a series of immune infiltrating cells and immune pathways. So far as we know, this is the first study to demonstrate the genomic profile, prognostic significance, and potential immune implication of *METTL5* in LUAD.

In LUAD, genes such as ROS1, RET, EGFR, and ALK have been found to be related to the development and progression, and targeted drugs can improve the prognosis of patients (Ou et al., 2014; Lee et al., 2015; Mazières et al., 2015; Lin J. J. et al., 2016). However, the clinical and prognostic effects of 18srRNA methylation-related genes are poorly understood. At present, the research on the *METTL5* mainly focused on its importance in the development of the nervous system (Liu et al., 2020), and its related effects on tumors have never been reported. Through previous studies, it has been found that the *METTL5* gene regulates the m6A methylation modification of 18sr RNA to regulate the expression of multiple genes (Van Tran et al., 2019). Encouragingly, compared with high *METTL5* expression, low *METTL5* expression was highly correlated with more OS in patients with LUAD. Further analyses suggested that *METTL5* was an independent prognostic factor and its prognostic value was not affected by tumor stage.

Multi-omics analysis provides some clues to explore the mechanism of *METTL5* gene overexpression. The methylation level of *METTL5* gene 1st Exon and TSS1500 in tumor tissues is significantly lower than that in normal lung tissue, which suggests that these two sites may involve in the aberrant expression of METL5. Combining the results of the *METTL5* gene RNA-seq with gene mutation and CNV data, the results suggested that *METTL5* gene mutations were not common in tumor tissues, and CNV amplification might play a role. Although previous studies have found that the amplification of some genes in tumors is not significantly related to their high expression (Caburet et al., 2015), overexpression of specific genes may be caused by CNV amplification in tumors (Ohshima et al., 2017). This suggests that CNV amplification and methylation may be a potential regulatory mechanism of *METTL5’*s overexpression.

Since prediction power of single gene was still insufficient, in order to further optimize the prediction power, we further screened four genes that have great correlation with the *METTL5* gene and have predictive value for tumor prognosis through multiple machine learning algorithm. These four genes formed a signature together with *METTL5* and were further validated in independent cohorts. The results proved the importance of MAPS as an independent prognostic factor in three cohost.

Through analysis, we found that patients with low MAPS scores, such as CTLA-4 and TCR, are more enriched in genes related to immune recognition pathways; while patients with higher MAPS scores are more enriched in proto-oncogene-related pathways such as P53 and RB. Through functional enrichment analysis, we found that the MAPS score could reflect the immunological characteristics of tumor tissues to some extent. The activation of immune-related pathways was considered to be related to a better prognosis (Fong and Small, 2008; Callahan et al., 2010; Mizuno et al., 2019). In patients with high MAPS, the enrichment of P53 and RB-related pathways suggested that this group of patients may have a higher mutation background, which in turn was considered to be benefit from immunotherapy. In addition, we further analyzed the differences of immune infiltrating cells and TMB, which was currently recognized as immunotherapy markers, between the two groups of patients. Through further analysis of tumor infiltrating immune cells, it could be found that in patients with high MAPS presented significant lower abundance of CD4+ and CD8+ T cells infiltrate compared with high-risk group, and these cells have also been proved to be closely related to tumor prognosis (Santarpia and Karachaliou, 2015; Mittal et al., 2016; He et al., 2017; Altorki et al., 2019). Patients in low MAPS owned high abundances of immune infiltrating cells, suggesting a more active immune response, which may be one of the reasons for the better overall prognosis of these patients. In addition, our analyses also reported a significantly positive correlation between MAPS and TMB, which suggested patients with high risk score may be benefited from immunotherapy using immune checkpoint inhibitors (Dong et al., 2017; Schrock et al., 2017). In summary, patients with high MAPS may own a higher mutational load and immunogenicity, and their anti-tumor immune cell infiltrations were suppressed. Thus, this subgroup of patients may benefit from immune checkpoint inhibitors and extend overall survival.

It is worth noting that there were three unavoidable limitations in our study. First of all, due to the limited number of patients with stage IV LUAD in the TCGA database, it was impossible to verify the relationship between *METTL5* expression and the prognosis of patients with stage IV LUAD. Secondly, it was rare for patients receiving immune checkpoint inhibitor therapy with complete transcriptome information, so the relationship between MAPS and immunotherapeutic response cannot be fully evaluated. Finally, although we initially explored the biological characteristics of *METTL5* in LUAD through enrichment analysis, the detailed mechanism of *METTL5* and LUAD progression, metastasis, and immune microenvironment still needed further biological experiments.

In summary, multi-omics profile of *METTL5* demonstrated significant variation between LUAD and normal lung tissue. The overall survival of patients with low *METTL5* expression is better than that of patients with high expression regardless of tumor stage. A robust prognostic signature named MAPS was constructed based on *METTL5* and the predictive performance was further validated in three independent cohorts. In addition, patients with high MAPS may be benefited from immunotherapy.

## Data Availability Statement

Publicly available datasets were analyzed in this study. This data can be found here: The datasets collected in the current study are available in the TCGA^3^ and GEO repository^4^.

## Author Contributions

YG and JH directed and designed the study. GZ, JW, YY, WG, and ZY collected and tidied the data. SS and KF performed the data analysis. SS wrote the manuscript. JW and QX reviewed and edited the manuscript. All authors read and approved the manuscript.

## Conflict of Interest

The authors declare that the research was conducted in the absence of any commercial or financial relationships that could be construed as a potential conflict of interest.

## Footnotes

1 https://portal.gdc.cancer.gov/

2 https://www.proteinatlas.org/

3 https://portal.gdc.cancer.gov/

4 https://www.ncbi.nlm.nih.gov/geo/

## Supplementary Material

Supplementary Figure 1 Kaplan–Meier survival curves and univariate Cox regression of different LUAD subtypes. The adenomas, acinar, and cystic represented adenomas and adenocarcinomas, acinar cell neoplasms and cystic, mucinous, and serous neoplasms, respectively.

Supplementary Table 1 The top 100 *METTL5*-associated genes identified by Spathial algorithm.

Supplementary Table 2 The full list of significantly enriched pathway between two risk groups.
