# Supplementary figures and images for: Construction and Comprehensive Analyses of a METTL5-Associated Prognostic Signature With Immune Implication in Lung Adenocarcinomas
Source: Front Genet. 2021 Feb 19;11:617174. doi: 10.3389/fgene.2020.617174 (PMC7933593; doi:10.3389/fgene.2020.617174)

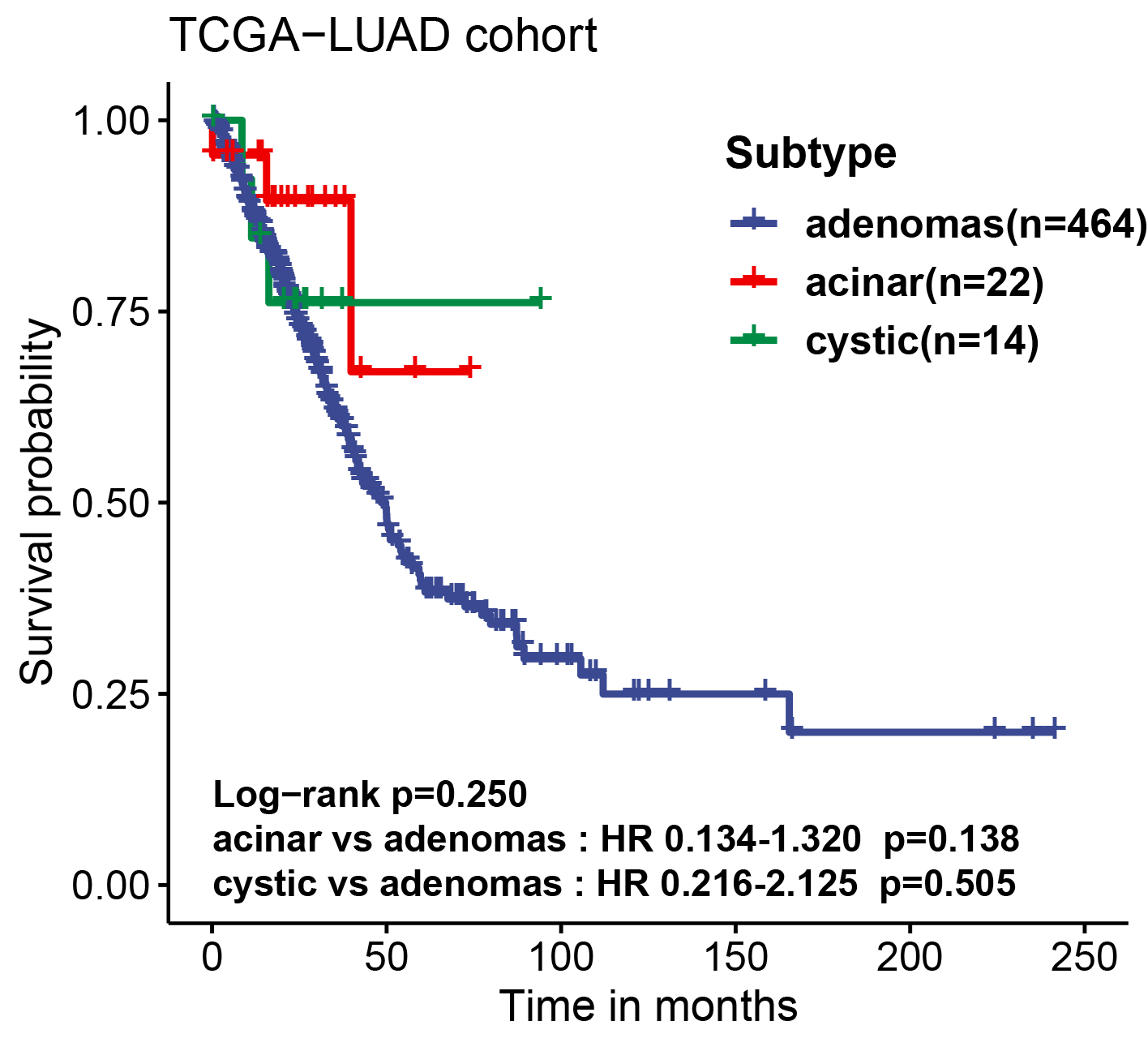

Supplement: Supplementary Figure 1 — Kaplan–Meier survival curves and univariate Cox regression of different LUAD subtypes. The adenomas, acinar, and cystic represented adenomas and adenocarcinomas, acinar cell neoplasms and cystic, mucinous, and serous neoplasms, respectively. [file Image_1.TIF]
